# Screening of poly-beta amino ester coated emulsion of ketorolac for cartilage delivery†

**DOI:** 10.1039/d4tb00313f

**Published:** 2024-05-28

**Authors:** Tahani Saeedi, Polina Prokopovich

**Affiliations:** a Cardiff School of Pharmacy and Pharmaceutical Sciences Cardiff UK prokopovichp@cf.ac.uk

## Abstract

Osteoarthritis (OA) is a prevalent chronic health condition necessitating effective treatment strategies. Globally, there were 86 million people with incident knee osteoarthritis in 2020. Pain management remains the primary approach to OA as the nature of cartilage poses challenges for drug delivery. An emulsion-based delivery system, using a class of positively charged and hydrolysable polymers (poly-beta-amino-esters) to coat oil droplets containing drugs, has been shown to enhance and prolong drug localization in *ex vivo* cartilage models. As the properties of the polymers used in this technology strongly depend on the monomers used in the synthesis, this study presents the screening of a wide range of PBAEs as droplet coating agents and using ketorolac as a model of nonsteroidal anti-inflammatory drugs. The emulsions prepared with this PBAE library were characterized, and drug localisation and retention were evaluated in both native and glycosaminoglycan (GAG) depleted cartilage *ex vivo* models. Optimal candidates were identified and tested in an *ex vivo* model showing the ability to protect chondrocyte cell viability and increase both GAG and collagen contents in cartilage exposed to cytokine (IL-1α) simulating acute cartilage damage. This work demonstrates the potential of PBAE coated emulsion as a delivery system for effective drug delivery in OA treatment.

## Abstract 2

Osteoarthritis (OA) is a prevalent chronic health condition necessitating effective treatment strategies.

## Graphical abstract

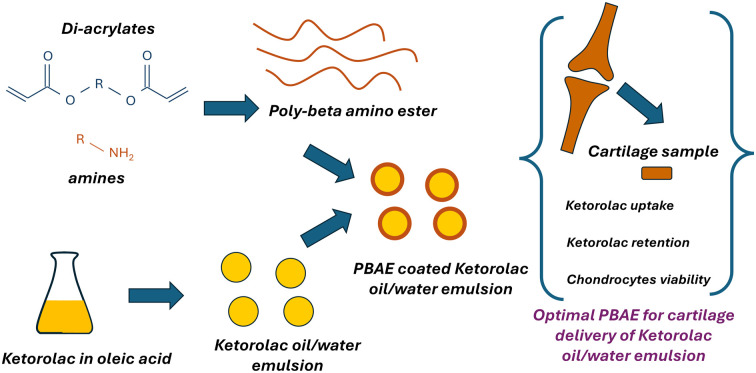

## 1 Introduction

Osteoarthritis (OA) is a chronic health condition and the most common form of arthritis; it is characterised by the gradual loss of articular cartilage leading to difficulty in joint movement, stiffness and moderate to severe pain.^1,2^ It can affect knees, hands, hips, feet, fingers and many other different parts of the body.^2^ Current treatments consist of physical therapy, life-style changes, oral medications, intra-articular injections and surgical treatment, with the main aim being pain management, while improving function and quality of life.^3^ Nevertheless, effectively delivering therapeutics and agents directly to the affected cartilage is challenging. This is mainly due to the cartilage avascular structure with a small pore size (<15 nm) and a highly anionic matrix composed of negatively charged proteoglycans.^4^ All these characteristics influence the ability of small molecule drugs to localise in articular cartilage and accelerate clearance from affected joints.^4,5^ It is suggested that intra-articular administration of OA drugs can offer potential treatment benefits due to highly therapeutic concentrations in joints and a decrease in systemic bioavailability that is associated with oral administration and could lead to certain side effects;^6^ however, drugs delivered intra-articularly have only a short retention time inside joints (hours to days).^7–10^

Non-steroidal anti-inflammatory drugs (NSAIDs) are widely known and the preferred medicines for the treatment of OA pain and inflammation.^11–14^ Although NSAIDs have significant therapeutic outcomes, they also cause gastrointestinal and cardiovascular side effects which are mainly associated with the oral route of administration. Additionally, frequent dosing is required with NSAIDs which may lead to patient non-compliance. The dominant hydrophobic nature of these compounds also leads to reduced residence time inside the affected tissues and rapid diffusion into the systemic circulation.^15,16^

Improving the solubility of NSAIDs and using polymers as drug delivery systems are essential when using these drugs to target cartilage in OA joints.^14^ Drug-delivery technologies are needed to reduce administration frequency and allow sustained drug release^17^ and drug encapsulation is one of the delivery strategies known to effectively achieve this.^18^

Poly-beta-amino-esters (PBAEs) are copolymers of an amine and a di-acrylate,^19,20^ and they have been developed for gene delivery as they bind to negatively charged strands of DNA, thereby allowing release into cells.^21^ Different approaches have been developed for DNA delivery, these include the use of viral and non-viral systems. Non-viral delivery systems are generally not as efficient as viral vectors but are considered safer, more cost-effective and less immunogenic;^22^ among the non-viral systems, PBAEs are highly customisable and, compared to the commonly used positively charged polymers (poly(l-lysine) (PLL) and polyethylenimine (PEI)), are not hampered by toxicity.^23,24^ They have also shown positive results when used as drug delivery systems for cartilage by directly conjugating corticosteroids to the polymer;^8–10^ PBAEs have also supported the delivery of NSAID emulsion droplets^14^ or licofelone^25^ in cartilage tissue. These applications driving cartilage drug localisation are based on the electrostatic interaction between the positively charged PBAE polymers and the negative charged glycosaminoglycans (GAGs) present in cartilage tissue.^7,8^ Furthermore, because the properties of the polymer depend on the monomers constituting the PBAE backbone, the efficacy of drug delivery systems using PBAEs is determined by the amine and the di-acrylate used.^9,10^

This study aims to optimise the PBAE used to coat a novel emulsion system prepared by encapsulating ketorolac within an oleic acid emulsion in order to ascertain the full potential of such approaches. The objectives of this study were firstly to synthesise a large library of PBAEs and use them to identify potential candidates through the investigation of the ability of PBAE coated emulsion containing ketorolac to deliver and retain such drugs in cartilage tissue. Secondly, to confirm the ability of the most promising PBAE coated emulsion candidate to inhibit cytokine-induced degradation of the ECM, to support the recovery of cell biosynthesis rates and prevent cell viability loss in an *ex vivo* OA model.

## 2 Experimental

### 2.1 Chemicals

All amine (piperazine, *N*,*N* bis [3-(methylamino)propyl] methylamine, and dimethylamino-1-propylamine) and acrylate (1,4 butanediol di-acrylate, 1,6 hexanediol di-acrylate, neopentyl glycol di-acrylate, 1,3 butanediol di-acrylate, and bisphenol A ethoxylate di-acrylate) compounds for the synthesis of PBAEs, sodium acetate, and ketorolac were purchased from Sigma, UK.

The solvents used for polymer synthesis and HPLC mobile phase (dichloro-methane, diethyl-ether, acetonitrile, and acetic acid glacial), as well as PBS, were purchased from Fisher, UK.

All chemicals were used as received and stored as recommended by the manufacturer.

### 2.2 PBAE synthesis

**Fig. 1:**
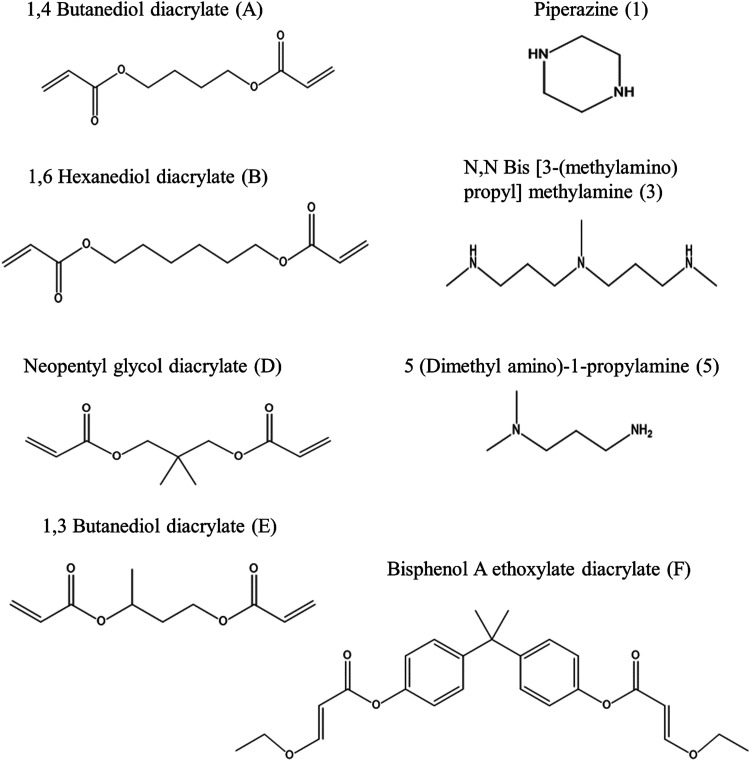
PBAE acrylates, amine names, chemical structures and coding systems of compounds used to prepare different types of poly-beta amino ester polymers.

15 different types of amino-terminated PBAEs were synthesised according to Michael addition reaction by the copolymerization of a specific di-acrylate monomer and either a secondary or tertiary amine monomer in a 1 : 1.1 ratio;^9,20^ each PBAE is abbreviated in the text using a letter to describe the di-acrylate and a number to represent the amine (Fig. 1) used for the preparation. For example, B5 was prepared by mixing 4 mmol of 1,6 hexanediol diacrylate with 4.4 mmol of dimethylamino-1-propylamine. Then, 5 ml of dichloromethane (DCM) was added to the mixture; polymerisation was performed at 50–55 °C for 48 hours.^8,20,26^ Then, approximately ten times the volume of diethyl ether (Fisher) was added (∼50 ml) to the mixture after it reached room temperature to recover the polymer through precipitation. After this, it was centrifuged at least three times for 5 minutes at 1300 rpm and washed with diethyl ether. The supernatant was removed every time, and finally, the polymer was dried using a rotatory evaporator.^8,20,26^ The newly synthesised polymer was isolated as a clear viscous liquid (A5, B5, D5, D1 and F1), yellowish sticky liquid (F3, F5, A3, D3, B3 and E3), white waxy block (for A1 and B1) or brown viscous liquid (E5 and E1). All PBAEs were kept in the fridge for no more two weeks before use.

### 2.3 Ketorolac and emulsion preparation

40 mg of ketorolac were mixed with 1 ml of oleic acid and stirred at 300 rpm with a magnetic stirrer until fully dissolved. Finally, 4 mg ml^−1^ ketorolac in the oleic acid emulsion was formulated by mixing 100 μl of the previous solution (40 mg ml^−1^) with 900 μl of PBS and stirring at 300 rpm overnight; throughout the text, this oil-in-water emulsion is referred to as an o/w emulsion.

PBAEs were dissolved in 100 mM acetate buffer pH 5 at a concentration of 2 mg ml^−1^.

The zeta potential of the PBAEs and the emulsions were measured separately. The amount of each PBAE required to fully coat the oil droplets was determined through the saturation method by adding PBAE to the emulsion in 10 μl aliquots and the zeta potential of the emulsion after each addition was measured; saturation was determined when the potential plateaued.^14,27^

### 2.4 PBAE characterisation

All PBAEs were characterised through ^1^H-NMR spectroscopy to assess purity after preparing samples 10–12 mg mL^−1^ in DMSO-d_6_.^28^

The zeta potential and hydrodynamic size (dynamic light scattering (DLS)) of PBAEs and emulsions were measured using a Malvern Zetasizer Nano ZS (Malvern Instruments, Malvern, UK).

Zeta potential (mV) was calculated from electrophoretic mobility using the Smoluchowsky equation. Each data point is presented as the average of three measurements on three independent emulsions.

### 2.5 Cartilage samples

Cartilage samples were extracted from 6 to 8 days old immature bovine steer feet purchased from local abattoirs (F DRURY & SONS Ltd).^7,8,14,29^ Metacarpophalangeal joints were exposed and samples were extracted using 5 mm diameter biopsy punchers (Kai medical) (Fig. 1).

Glycosaminoglycan (GAG) depleted samples (simulating early OA) were prepared by digesting the cartilage disc in 500 μl of trypsin 1 mg ml^−1^ (Gibco) in PBS (Oxoid) for 24 hours at 37 °C.

For the experiments that required cartilage tissue cultures, cartilage discs were extracted and weighted to normalize the data. The discs were equilibrated in serum-free medium (low-glucose DMEM, l-glutamine, 25 mM HEPES, and 110 mg l^−1^ sodium pyruvate) supplemented with 5 ml of 1% insulin-transferrin selenium, 5 ml (100×) of minimum essential medium nonessential amino acid (MEM NEAA) (all from Gibco by life technologies), 4 M proline (Aldrich), 20 mg ml^−1^ ascorbic acid (Fisher Scientific), 100 U ml^−1^ penicillin, 100 μg ml^−1^ streptomycin and 250 μg ml^−1^ amphotericin B (Sigma Aldrich) for three days prior to treatment at 37 °C in a 5% CO_2_ atmosphere. The serum-free medium formulation is essential to maintain the cultured cartilage explant's mechanical and biochemical properties.^30^

### 2.6 Cartilage digestion

The cartilage digestion buffer solution consisted of 300 mg of papain, 1 mM ethylenediaminetetraacetic acid (EDTA) and 2 mM dithiothreitol (DTT) added to 1 litre of a pH 6.8 phosphate buffer.^8–10^ After conducting uptake and retention experiments, the cartilage samples were incubated individually in 1 ml of digestion buffer at 55 °C for 24 to 48 hours until the cartilage disc was completely dissolved.

### 2.7 Drug uptake into cartilage

Cartilage discs were weighed and fixed to the bottom of the wells of a 96 well plate using a small amount of high vacuum grease paying attention keeping the superficial layer (cartilage side) up. Each well was filled with 100 μl of the PBAE ketorolac o/w emulsion and then incubated at 37 °C for the required time. After this, each sample was removed, washed with an extensive amount of water to remove any debris, placed in an Eppendorf tube containing 1 ml of digestion buffer. Experiments were performed 3 independent times with 4 replicates, while the samples were obtained from 3 different bovine feet. A comparison of the drug uptake was made between the PBAE–ketorolac o/w emulsion at a concentration of 4 mg ml^−1^ and either ketorolac prepared in PBS (P control) and the uncoated ketorolac o/w emulsion (E control).

### 2.8 Drug retention into cartilage

Cartilage samples were treated with the ketorolac o/w emulsion in 96 well plates for 10 min (as described before). Then, after washing with an extensive amount of water, they were incubated at 37 °C in an Eppendorf tube containing 500 μl of PBS for different periods of time. After this, the cartilage was removed, washed with water, and placed in 1 ml of digestion buffer. Experiments were performed 3 independent times with 4 replicates, and samples were obtained from 3 different bovine feet.

### 2.9 Drug quantification

Reverse phase-HPLC (Agilent 1220LC) was used to quantify the amount of ketorolac in the cartilage. An Agilent HPLC system was used with a Teknokroma TRACE EXCEL 120 ODSB 5 μm column at 25 to 30 °C.^14^ The amount of drug contained in the cartilage was expressed as the mass of drug per wet cartilage mass.

### 2.10 Effect of the drug delivery system on the IL-1α treated cartilage

#### 2.10.1 Treatment of cartilage with cytokine (IL-1α)

Cartilage explants were treated with 1 ng ml^−1^ of IL-1α to mimic the post-traumatic OA cartilage. Samples were treated with IL-1α (1 ng ml^−1^) (Sigma Aldrich) for 20 days in the presence of the 4 mg ml^−1^ ketorolac o/w emulsion or 4 mg ml^−1^ ketorolac o/w emulsion coated with the PBAE polymer. Pure media with or without IL-1α, along with the 4 mg ml^−1^ ketorolac o/w emulsion coated with the PBAE polymer without IL-1α were used as control groups. During the 20 days culture period, the medium for all treatment groups was replenished every 2 to 3 days.^4,14^

#### 2.10.2 Quantification of glycosaminoglycan (GAG)

DMMB (16 mg) was added to 1 l of dH_2_O containing 3.04 g of glycine, 2.37 g of NaCl and 95 ml of 0.1 M HCl. 40 μl of the digested cartilage explants were added into a 96 well plate in 5 replicates, followed by the addition of 200 μl of the DMMB reagent, and then the colour density was measured immediately at 525 nm.^31^ The GAG content was determined through a freshly prepared calibration curve using chondroitin 6-sulfate from shark cartilage (Sigma, UK) as standards.^29^

#### 2.10.3 Collagen content determination assay

The collagen content was measured using the hydroxyproline assay. 100 μl of the cartilage digest solution was aliquoted in Eppendorf tubes and hydrolysed using 500 μl of 12 N HCl at 100 °C for 18 h.^32^ After this, the hydrolysate was dried in an oven at 55 °C for 48–72 hours and the residue was dissolved in 150 μl of dH_2_O and then dried in a chemical hood for another 48–72 hours. Using a 96 well plate, 60 μl of dH_2_O were added to each well, followed by 20 μl of assay buffer (3 ml of 1-propanol, 2 ml of dH_2_O and 10 ml of pH 6 citrate buffer) and 40 μl of chloramine T solution (0.14 g of chloramine T, 500 μl of dH_2_O, 4 ml of pH 6 citrate buffer and 500 μl of dH_2_O). The reagent was then allowed to react for 15 minutes at room temperature. Finally, 20 μl of 1-propanol, 30 μl of perchloric acid and 30 μl of *N*,*N*-dimethylbenzylamine were added. The plate was left in a 70 °C oven for 20 mins. Finally, after allowing the plate to cool down, the colour density was measured at 570 nm using a plate reader.^32^

#### 2.10.4 Live/dead staining and imaging

The live/dead^TM^ viability/cytotoxicity kit (Invitrogen by Thermo Fisher Scientific) was used. The stain buffer was prepared by adding 1 μM calcein-AM and 2 μM ethidium homodimer-1 to 10 ml of sterile PBS; the solution was used immediately.

On the day of the experiment, 100–150 μm thick cartilage slices were cut from the centre of cartilage discs using a sharp-blade. Slices were immediately stained with 200 μl of live/dead staining solution for 30 minutes in the dark at room temperature. Slices were washed with PBS and imaged with a 10× objective lens using a LSM (Zeiss LSM880) (calcein AM excitation/emission ∼493–582 nm and ethidium homodimer excitation/emission ∼582–741 nm). Live chondrocyte cells were stained green with calcein-AM and dead cells were stained red with ethidium homodimer; the cells were imaged using a laser scanning confocal (LSM) microscope (Zeiss LSM880).

#### 2.10.5 XTT assay

Cartilage tissue viability was analysed using a cell proliferation assay kit II (XTT) (Roche) with a small adaptation to the company's instruction. In brief, pre-weighed cartilage tissues were cultured in media for 20 days. Then, the cartilage disc was cut using a scalpel into approximately 4–6 pieces and incubated in the XTT solution (1 ml) for 4 hours at 37 °C. Then, the XTT solution was removed and retained to be used later. After this, 0.5 ml of dimethyl sulfoxide (DMSO) was added to extract the tetrazolium product from the tissues and incubated for 1 hour. Prior to measuring the absorbance, the XTT and DMSO solutions were mixed appropriately, and then the absorbance of the samples was measured using a microplate reader in triplicate at 450 nm and 690 nm (Tecan, Infinite 200 PRO) in a 96-well plate. Finally, the absorbances at 690 nm were subtracted from those at 450 nm, and the XTT content was calculated per sample weight.

#### 2.10.6 Histological evaluation of cartilage tissues

The cartilage slices were fixed in 10% (v/v) neutral buffered formalin (Sigma-Aldrich) for 48 hours. Then, samples were dehydrated through increasing ethanol concentrations (70–96%) for 1 min, followed by xylene for 4 min and then embedded in paraffin wax for 7 days. After this, serial sections (5 μm) were rehydrated, and stained with Weigert's haematoxylin (Atom Scientific) for 3 min, differentiated in 1% (v/v) acid–alcohol (Sigma-Aldrich), stained with 0.02% (w/v) aqueous fast green (Sigma-Aldrich) for 5 min and washed briefly in 1% (v/v) acetic acid before staining with 0.1% safranin O (Sigma-Aldrich) for 5 min.

Finally, sections were dehydrated and mounted. Images were captured using a Leica DMRB photomicroscope controlled by Zen Pro software 2012 (Zeiss).

### 2.11 Statistical analysis

After data were assessed for normality, statistically significant differences between the mean of ketorolac uptake, retention, GAG and the collagen content were tested using paired sample *t*-test or one-way ANOVA with a Tukey's *post hoc* test (*p* < 0.05). The statistical analysis was performed using IBM SPSS Statistics (Version 25).

## 3 Results

### 3.1 Zeta potential measurements

**Fig. 2:**
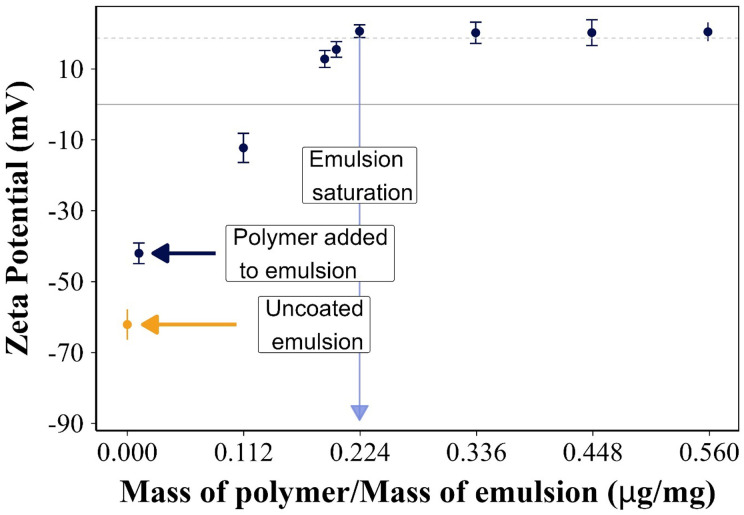
Zeta potential (mV) value of ketorolac emulsions during coating with B5 until saturation (mean ± SD, *n* = 3).

Uncoated emulsions exhibited a negative charge and the addition of PBAE gradually increased the charge of the droplets up to a zeta potential similar to that of the pure PBAE used to coat the oil droplets; this occurred when the surfaces of the drops were saturated by the coating electrolyte (Fig. 2). The polymer interacted differently with the ketorolac o/w emulsion in terms of an amount required for complete coating (Table 1); for example, 80 μl of B5 (equivalent to 0.224 μg of the polymer per mg of emulsion) were needed (Fig. 2), while 1800 μl of F1 were required to completely coat the ketorolac o/w emulsion and to change its negative charge from −73.9 mV to +23.1 mV.

**Table 1:** Characterisation of the coated emulsion – zeta potential (mV) values for ketorolac emulsions before and after coating with PBAEs and the amount of each PBAE required to coat the ketorolac emulsion completely

PBAE	Zeta potential (mV) of polymer alone	Zeta potential (mV) of PBAE coated ketorolac emulsion	Saturation – volume (μl) of PBAE (2 mg ml^−1^) added to 1 ml of emulsion (10% w/w)
Uncoated emulsion	−73.9 ± 0.5		
A1	+12.3 ± 3.5	+13.3 ± 2.3	1500
B1	+11.4 ± 1.4	+18.4 ± 0.8	1300
D1	+14.1 ± 1.6	+5.7 ± 1.3	1000
E1	+13.8 ± 3.8	+12.6 ± 0.7	1500
F1	+13.9 ± 8.5	+23.1 ± 0.5	1800
A3	+15.4 ± 1.5	+33.3 ± 1.9	360
B3	+16.8 ± 2.9	+31.8 ± 2.6	650
D3	+17.1 ± 1.4	+39.2 ± 3.9	600
E3	+11.3 ± 0.7	+42.2 ± 1.6	650
F3	+19.0 ± 5.5	+39.6 ± 2.3	650
A5	+12.6 ± 0.4	+17.1 ± 1.9	160
B5	+18.7 ± 0.9	+20.2 ± 3.6	80
D5	+9.7 ± 0.4	+9.3 ± 0.7	180
E5	+7.3 ± 0.7	+8.6 ± 1.4	500
F5	+12.9 ± 0.3	+18 ± 4.3	500

### 3.2 Droplet size

The initial particle diameter of the ketorolac o/w emulsion was 1.50 ± 0.02 μm. As shown in Table 2, coating the ketorolac o/w emulsion with A1, B1, and F5 led to ten fold increase in the ketorolac particle size, while the increase was 20 times when coated with E1, B3, A5, and B5. In contrast, using F1 and E5 to coat the ketorolac emulsion led to its size increasing 4 times with the difference being statistically significant (*p* > 0.05).

**Table 2:** Size measurements (μm) for ketorolac emulsions coated with 15 different types of poly-beta amino ester polymers

PBAE	Droplet diameter (μm) after PBAE saturation
Uncoated ketorolac emulsion	1.50 ± 0.02
A1	2.13 ± 0.20
B1	2.92 ± 0.19
D1	3.46 ± 0.57
E1	4.53 ± 0.31
F1	0.70 ± 0.06
A3	1.03 ± 0.08
B3	4.01 ± 0.19
D3	1.53 ± 0.15
E3	1.46 ± 0.06
F3	1.85 ± 0.18
A5	2.00 ± 0.08
B5	2.30 ± 0.40
D5	3.23 ± 0.90
E5	0.72 ± 0.09
F5	2.24 ± 0.30

### 3.3 Drug uptake measurements

**Fig. 3:**
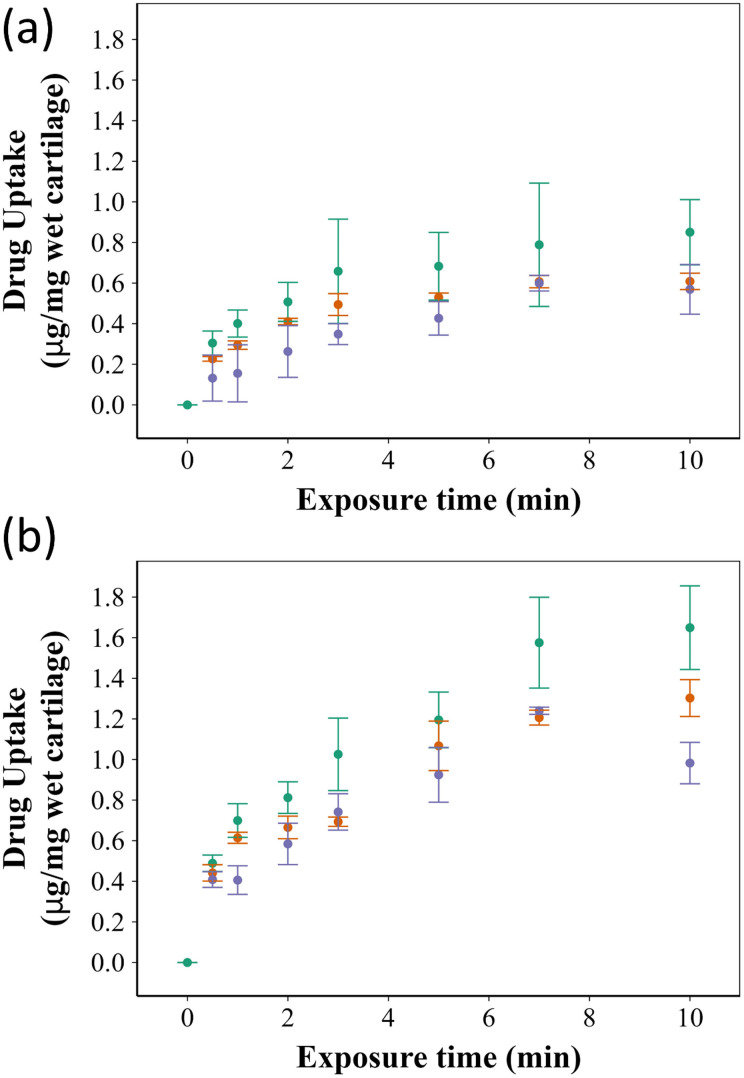
Comparison of ketorolac uptake in untreated (A) and GAG depleted (B) cartilage using 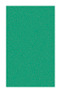 the ketorolac o/w emulsion coated with B5, 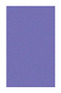 ketorolac in PBS, and 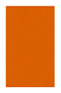 the uncoated ketorolac o/w emulsion. (Mean ± SE, *n* = 3).

Fig. 3 shows the cartilage uptake of ketorolac after 10 min exposure to the B5 coated ketorolac o/w emulsion compared to ketorolac alone (P control) and the ketorolac o/w emulsion with the difference being statistically significant (*p* < 0.001).

The amine constituents of the polymer backbone impacted drug uptake, the most effective was *N*,*N*-bis 3-(methylamino) propyl methylamine, followed by 3-(dimethyl-amino)-propylamine and piperazine. Generally, the presence of additional nitrogen atoms in the amines increased the positive charges of the polymer chain, leading to an increase in electrostatic attraction between the negatively charged polymers and the extracellular cartilage matrix. When the cartilage was exposed to ketorolac at equal concentrations either the commercial formulation of ketorolac, ketorolac o/w emulsion, or the coated form with PBAE, the ketorolac o/w emulsion coated with PBAE resulted in a higher amount of the drug in the cartilage even after a very short time (1 min). Overall, all 15 types of PBAE examined were similarly effective in increasing ketorolac uptake inside the cartilage tissue, especially in GAG depleted samples and in comparison to the controls (Fig. S1, ESI†).

### 3.4 Drug retention measurements

**Fig. 4:**
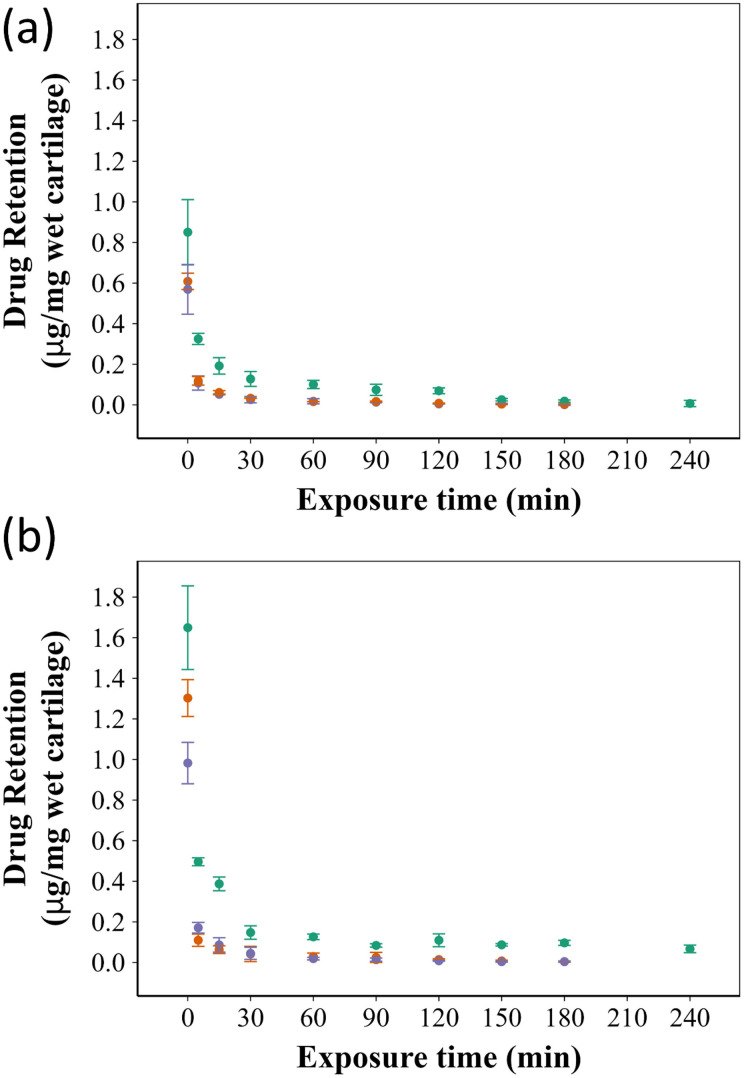
Comparison of ketorolac retention in both untreated (A) and GAG depleted (B) after 10 min exposure to 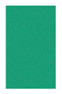 the ketorolac o/w emulsion coated with B5, 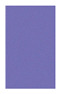 ketorolac in PBS, and 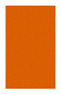 the uncoated ketorolac o/w emulsion (mean ± SE, *n* = 3).

Fig. 4 shows a comparison between ketorolac retention in the untreated cartilage and GAG depleted cartilage using the B5 polymer. Ketorolac was released quickly from the cartilage in the untreated sample, with the ketorolac concentration falling below detection limits after about 2 hours in the case of controls. However, when PBAE was used to coat the emulsion droplets, ketorolac was still detectable in the cartilage even after 5 hours. In the GAG depleted cartilage, release was slower than that in the untreated samples; yet, the amount of ketorolac left in the tissue using the ketorolac o/w emulsion coated with B5 was always higher than that for the controls. Overall, all 15 types of PBAEs considered effective in increasing the ketorolac retention time in cartilage tissues compared to either of the controls (P and E) (Fig. S2, ESI†).

### 3.5 XTT

**Fig. 5:**
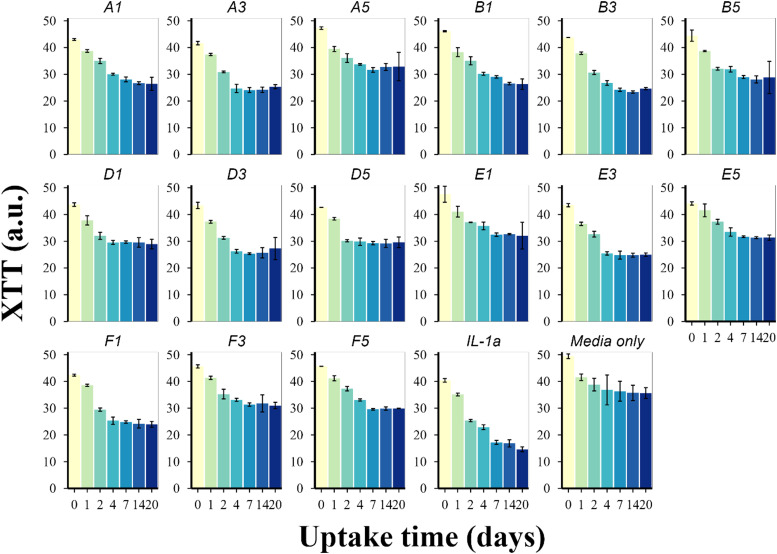
Viability assessment, through the XTT assay, of cartilage tissues over 20 days treated with components of the developed PBAE library (mean ± SD, *n* = 5).

The XTT assay indicated that from day 0 to day 20 the cartilage cell viability decreased when treated with IL-1α (*p* < 0.01), compared to the untreated control. Adding PBAE to the cartilage media, the cell viability was maintained compared to samples treated without PBAE (Fig. 5). These results showed that PBAEs are not toxic to the cartilage tissue as it maintained its viability compared to IL-1 α treated samples. A5, B5, E1, F3, and E5 were most effective in preserving high amounts of XTT in cartilage samples, indicating that the cartilage cell viability is similar to the untreated control over 20 days (Fig. 5). Furthermore, the difference of the XTT assay results at day 20 between samples exposed to B5 and the control was not statistically significant (*p* < 0.05).

### 3.6 Bovine *ex vivo* model

The optimal PBAE candidate was selected employing a multi-criteria decision approach using the results of the previous experiments using the 15 different types of PBAE polymers giving equal weight to all of the assay parameters (XTT, uptake and retention) considering the percentage of each parameter compared to the maximum observed.^33^ B5 was chosen for efficacy testing using an IL-1α exposure *ex vivo* model.

#### 3.6.1 Glycosaminoglycan (GAG) loss measurements

**Fig. 6:**
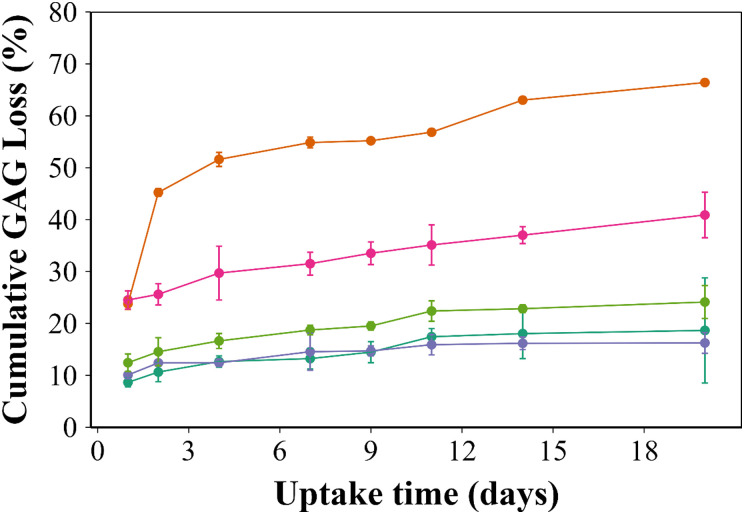
Quantification of cumulative sGAG loss, using the DMMB assay, in the cartilage exposed for 20 days to 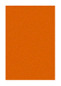 IL-1α, 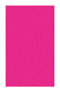 the uncoated ketorolac emulsion + IL-1α, 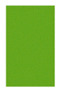 the B5 coated ketorolac emulsion + IL-1α, 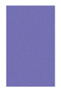 the B5 coated ketorolac emulsion and 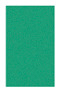 pure media only (mean ± SD, *n* = 5).

Cartilage samples cultured in media showed a slight decrease in the sGAG content over a period of 20 days (18.6 ± 10%) (Fig. 6). When IL-1α was added to the media, progressive ECM degradation was observed and more than 50% of the initial sGAG was lost after 72 hours. The simple addition of the ketorolac o/w emulsion coated with B5 to the media containing IL-1α reduced the inhibitory activity of IL-1α, with the difference being statistically significant (*p* <0.001). After 20 days, the untreated cartilage samples had lost around 17% of the sGAG content, compared to 66% in the IL-1α treated cartilage. Moreover, when the IL-1α treated cartilage was exposed to B5 with/without ketorolac, a remarkable decrease in sGAG loss was observed (*p* < 0.001). The percentage of sGAG loss was 3 to 5 times higher in the IL-1α treated cartilage than the untreated group (in media only) or samples exposed to the ketorolac o/w emulsion coated with B5 (*p* < 0.001) (Fig. 6).

#### 3.6.2 Collagen content determination

**Fig. 7:**
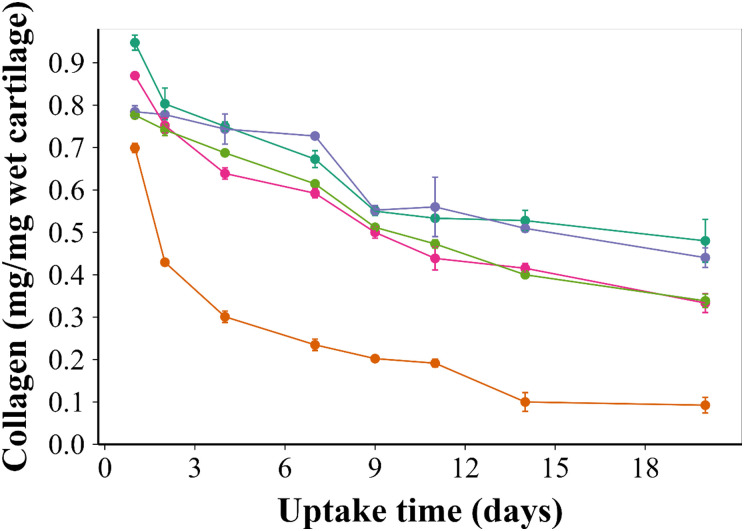
Quantification of collagen, using hydroxyproline assay, in the cartilage exposed for 20 days to 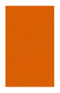 IL-1α, 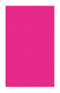 the uncoated ketorolac emulsion + IL-1α, 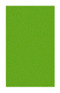 the B5 coated ketorolac emulsion + IL-1α, 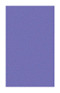 the B5 coated ketorolac emulsion and 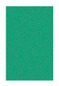 pure media only (mean ± SD, *n* = 5).

Cartilage sample cultures in medium containing IL-1α showed a 50% drop in the collagen content during the first week. This was followed by a steady decrease for 2 weeks, which was significantly lower (*p* < 0.01) than untreated cultured samples (*p* < 0.05). Overall, collagen degradation was significantly higher in samples exposed to IL-1α than any of the controls (*p* < 0.01) (Fig. 7).

Treating cartilage explants with the developed drug delivery system was significantly more effective in increasing collagen synthesis to the same level as the untreated control (*p* < 0.01) over 20 days (Fig. 7).

#### 3.6.3 Live/dead viability assay

**Fig. 8:**
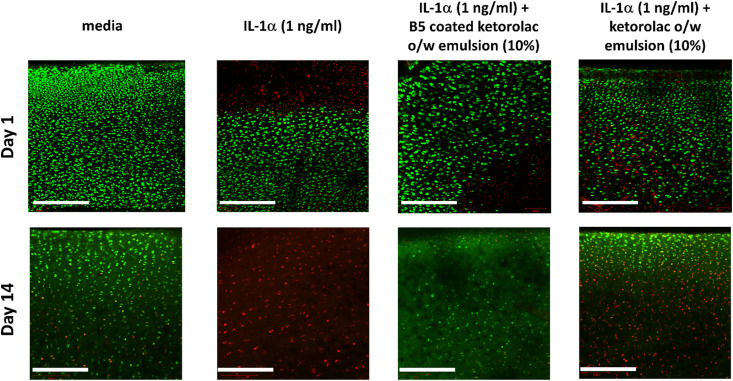
Examples of images of live/dead fluorescently stained bovine explants exposed to IL-1α (1 ng ml^−1^) to evaluate chondrocyte viability on day 1 and day 14. Live cells were stained green by calcein, and dead cells were stained red by ethidium homodimer. The scale bar is 300 μm.

Fluorescence images of the untreated cartilage sample showed viable chondrocytes (green) without the appearance of dead cells (red); only the addition of IL-1α resulted in dead cells clearly being observable close to the interface between the cartilage and the fluid after 1 day of incubation (Fig. 8). By day 2, the images of the tissue samples subjected to IL-1α showed a mixture of green and red fluorescence with a substantially higher proportion of red fluorescence than day 1 (Fig. S3, ESI†). After 14 days of incubation, the tissue had undergone significant changes as most of the chondrocytes in the IL-1α treated sample appeared dead as implied by the predominant red fluorescence (Fig. 8). The addition of the ketorolac o/w emulsion coated with B5 maintained chondrocyte viability during the 14 days of the incubation.

#### 3.6.4 Histological analysis of cartilage explants

**Fig. 9:**
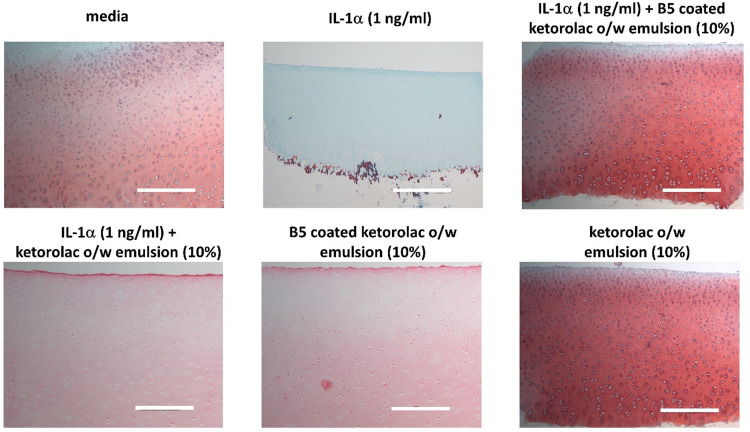
Histology sections of the cartilage tissue stained with safranin O on day 20 exposed to pure media, IL-1α (1 ng ml^−1^), the ketorolac o/w emulsion coated with B5. The reddish orange colour intensity indicates the amount of GAG in the sample. The scale bar is 300 μm.

Images of sections of the tissue from the cartilage cultures stained with safranin O are shown in Fig. 9. Images of untreated (media only) tissue sections revealed high GAG staining intensity on day 20. In the cartilage tissue treated with IL-1α, there was a significant reduction in staining intensity compared to the untreated tissue at day 20, indicating a massive loss of GAG from the tissue. Exposing samples to IL-1α along with the developed formulation containing B5 led to maintaining the GAG content to levels similar to those of the untreated cartilage samples (Fig. 9).

## 4 Discussion

PBAEs are biodegradable and hydrophilic, which makes them superior to other polycationic polymers and thus suitable for widespread use as drug delivery systems.^34^ Coating the ketorolac o/w emulsion with PBAEs resulted in augmenting the electrostatic interactions of the droplets containing the drug with the negatively charged GAG chains in the cartilage ECM. This electrostatic attraction resulted in higher drug uptake and prolonged retention times (Fig. 3 and 4); PBAE prepared using different amines and di-acrylate monomers had different properties and resulted in different levels of drug uptake/retention (Fig. 3 and 4), generally better than uncoated emulsions or ketorolac commercial formulation.

### 4.1 Structure–function activity relationship of PBAEs

Previous results were obtained using A5;^14^ and different combinations using further di-acrylates and amines were tested here synthesising a library of PBAEs using 4 other different di-acrylates with different chain lengths and exhibiting branches or aromatic rings and two other amines representing cycle compounds, and different chain lengths or locations of tertiary amines.

PBAE positive charges at pH 5 are determined by the protonation of nitrogen atoms in the polymer backbone; thus, the presence of secondary and tertiary amines increased the polymer charge that is key to promoting efficacy in binding to the negatively charged cartilage ECM.^8–10,14,25^ The structure of di-acrylates was not expected to impact the zeta potential as all compounds tested presented only ether moieties as possible sites for hydrogenation. Amines 3 and 5 are tertiary amines while amine 1 is a secondary amine (Fig. 1) and all exhibited a high positive charge (up to 19 mV) (Table 1) and demonstrated effective drug uptake and retention inside the cartilage (Fig. 3 and 4). D5 and E5 polymers had the lowest positive charges (+9.7 mV and +7.3 mV, respectively) resulting in low uptake and retention of ketorolac inside the cartilage.

### 4.2 Uptake and retention of ketorolac inside the cartilage tissue

In this work, PBAEs of sizes 0.2–5.0 μm with a positive zeta potential of 10–19 mV have been found to provide an effective delivery system for OA. It is notable that these sizes and charges differ somewhat from other types of delivery systems. For example, PBAE-NPs as plasmid carriers are effective with sizes of 0.07–0.150 μm and positive charges between 5 and 10 mV.^9,10^ Meanwhile, an avidin carrier of size >0.01 μm is considered to be ideal for OA drug delivery.^7^ The size of the droplets developed (∼1.5 μm) in this work was greater than the ∼10 nm threshold reported previously;^35^ however, the diffusion into the cartilage of the coated emulsions and other large therapeutic agents (micelles, liposomes and other micro-scale delivery agents) has been attributed to the ability of these molecules/vesicles to move through the larger space between collagen fibrils.^14,36,37^

Improving drug uptake and retention time inside the cartilage tissue is considered the first step towards studying the ability of a drug delivery system effectiveness and thus these parameters were used during the initial screening of the PBAE library considered. Generally, drug uptake was significantly higher in the ketorolac o/w emulsion coated with PBAEs than in either type of control (commercial formulation and uncoated emulsion) (Fig. 3); similarly, the emulsions presented here were also able to sustain ketorolac retention for almost 6 hours, compared to about 2 hours for the controls (Fig. 4). Moreover, the impact of a reduced GAG content in the cartilage, that is normally associated with OA, was evaluated as the mechanism of action of the proposed drug delivery system relying on the presence of negatively charged GAG. PBAE coated emulsions were also effective in delivery and sustain ketorolac localisation in GAG depleted tissues showing the viability of the proposed technology in conditions more closely resembling a pathological status. The reduced electrostatic interactions resulting from the lower GAG content may be compensated by larger porosity in the tissue that facilitates the diffusion of the oil droplets.

### 4.3 XTT

Overall, PBAE displayed similar satisfactory cell viability to the pure media (Fig. 5); this was expected and a consequence of the hydrolysable ester bond coupled with the biocompatibility of the hydrolysed products. These results confirmed previous reports that PBAEs are suitable for cartilage drug delivery and considerably less toxic than currently available cationic polymers such as poly-(ethyleneimine) and poly-l-lysine.^20,38,39^

### 4.4 IL-1α assays

The efficacy of delivering ketorolac into the cartilage *ex vivo* model was tested using only the ketorolac o/w emulsion coated with B5 as this polymer was the most promising candidate identified in the previous steps.

Drug uptake does not guarantee biological activity, as the active molecule must be released from the oil droplet in order to provide anti-inflammatory activity.

The PBAE based delivery system developed in this work was effective in preventing GAG loss/ECM degradation as the GAG content in samples exposed to IL-1α was comparable to that of samples not exposed to IL-1α hence demonstrating that ketorolac remained active when delivered through the proposed system (Fig. 6–8).

Furthermore, the drug delivery system effectiveness in preventing collagen degradation when IL-1α was added to the culture medium (Fig. 7) serves to further highlight the improved drug localisation achieved through the presented delivery system in line with the reported efficacy of downregulating the oxidative stress to promote tissue regeneration.^40–42^

## 5 Conclusions

The rational design of PBAEs has been undertaken to maximise the effectiveness of OA drugs in treating joint diseases considering a multitude of parameters such as not only drug uptake/retention but also chondrocyte viability and post-delivery drug activity in preventing GAG and collage depletion caused by IL-1α.

This work has proven that a ketorolac (model NSAIDs) and oleic acid-based o/w emulsion coated with PBAEs could potentially provide an effective delivery system for OA drugs due to the electrostatic forces of attraction between the negatively charged GAGs in the articular cartilage and the positively charged polymer emulsion coating.

## Conflicts of interest

PP is named inventor in patents related to PBAEs use in drug delivery.

## Supplementary Material

TB-012-D4TB00313F-s001
