# Supplementary material for: Screening of poly-beta amino ester coated emulsion of ketorolac for cartilage delivery
Source: J Mater Chem B. 2024 May 28;12(24):5930–9. doi: 10.1039/d4tb00313f (PMC11186506; doi:10.1039/d4tb00313f)
Supplement: TB-012-D4TB00313F-s001 [file TB-012-D4TB00313F-s001.pdf]

Electronic Supplementary Material (ESI) for Journal of Materials Chemistry B.
This journal is © The Royal Society of Chemistry 2024

Screening of poly-beta amino esters coated emulsion of ketorolac for cartilage delivery

By

Tahani Saeedi [a], Polina Prokopovich [a,*]

Supplementary info

	Untreated cartilage tissue	GAG depleted cartilage tissue
A1	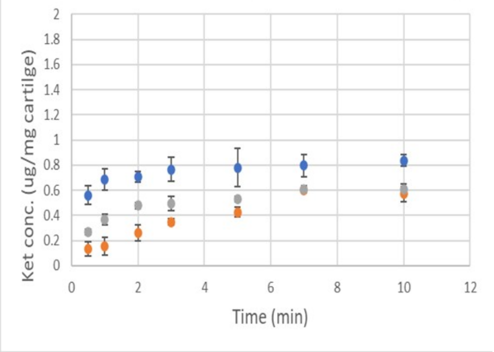	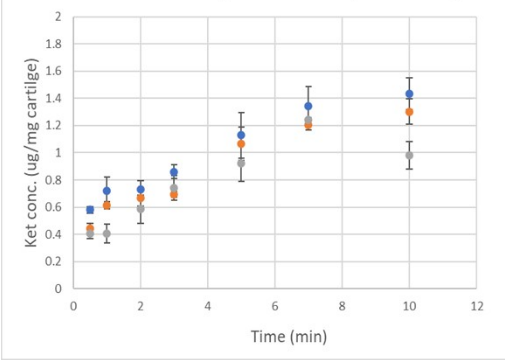
B1	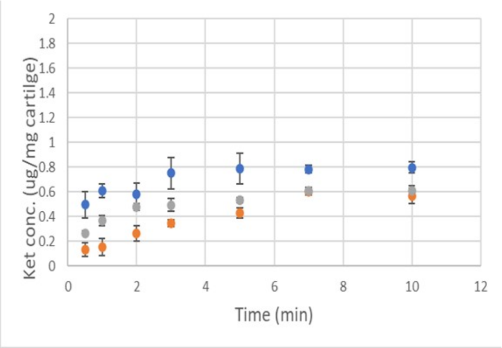	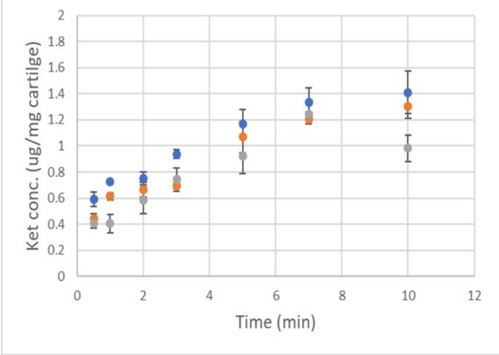
D1	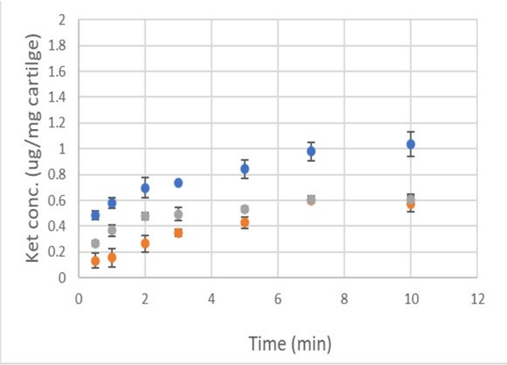	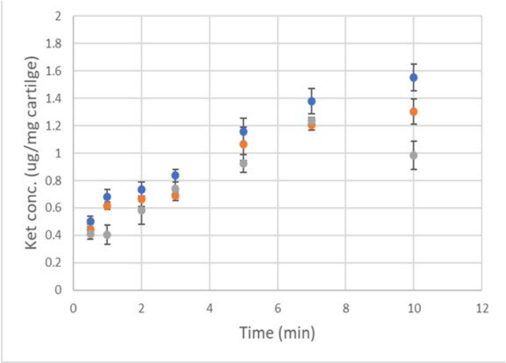
E1	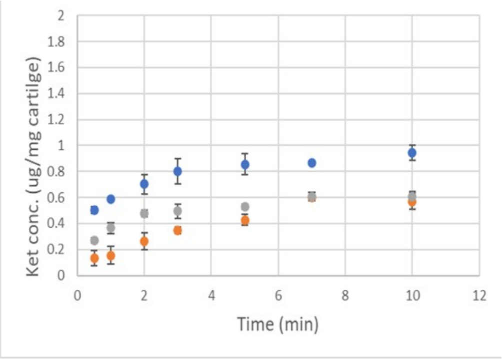	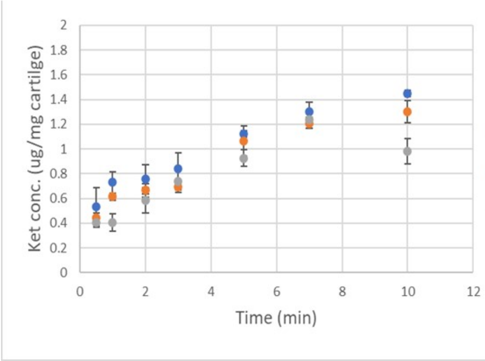

F1

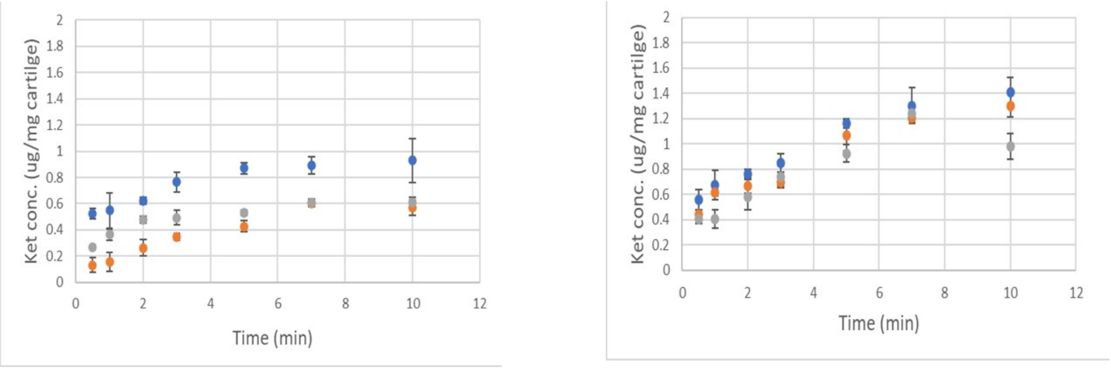

Ket conc. (ug/mg cartilge)
Time (min)

A3

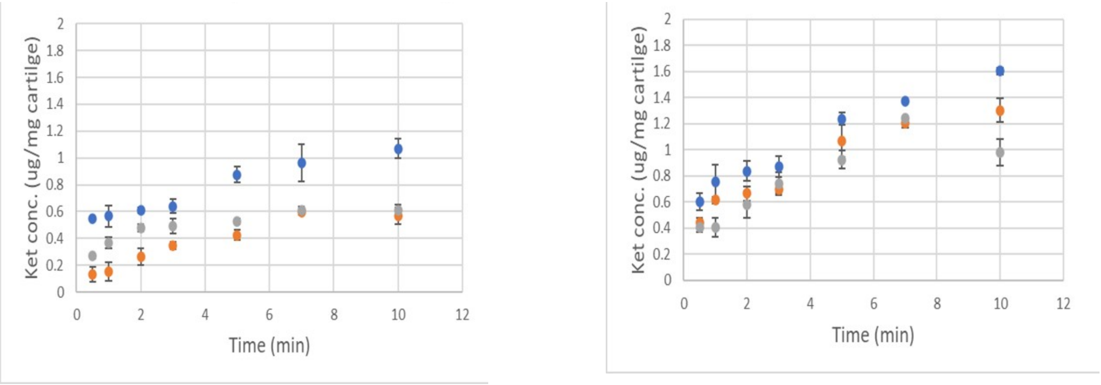

Ket conc. (ug/mg cartilge)
Time (min)

B3

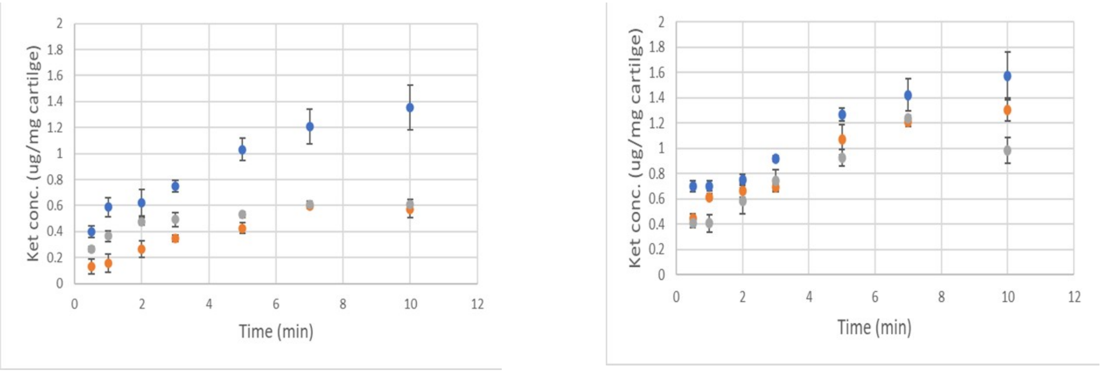

Ket conc. (ug/mg cartilge)
Time (min)

D3

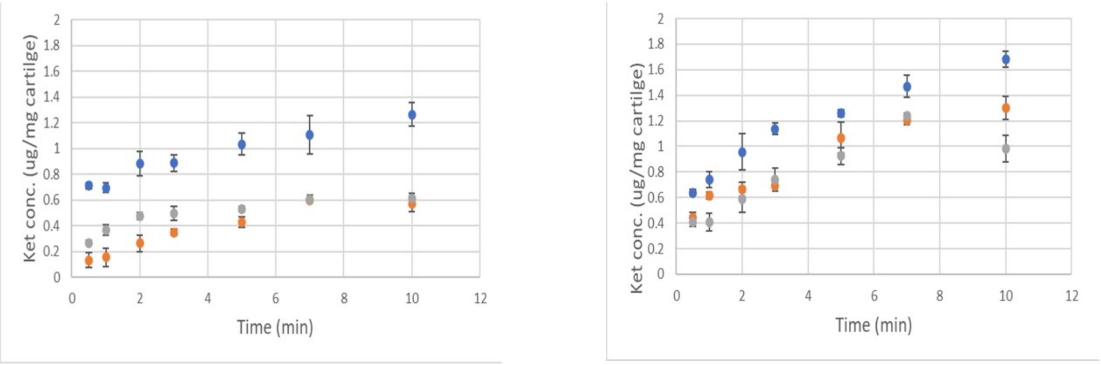

Ket conc. (ug/mg cartilge)
Time (min)

E3

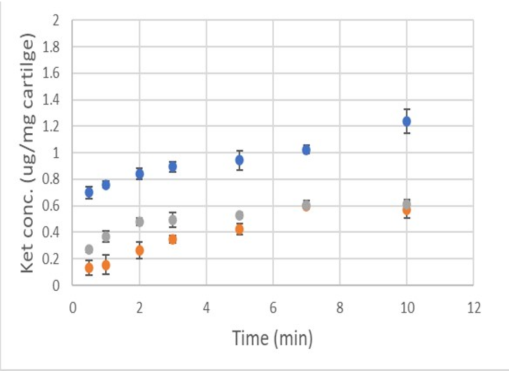
Ket conc. (ug/mg cartilge)
Time (min)

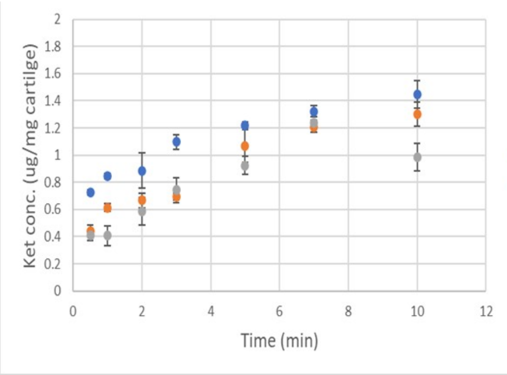
Ket conc. (ug/mg cartilge)
Time (min)

F3

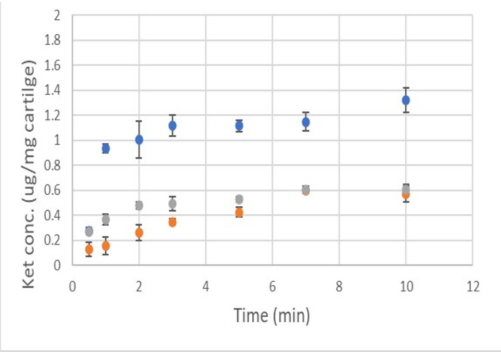
Ket conc. (ug/mg cartilge)
Time (min)

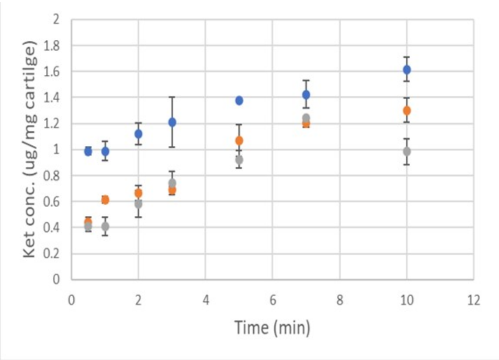
Ket conc. (ug/mg cartilge)
Time (min)

A5

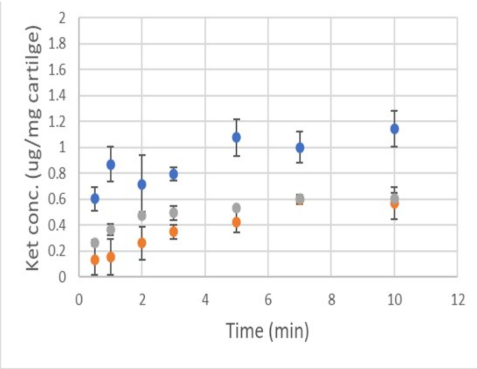
Ket conc. (ug/mg cartilge)
Time (min)

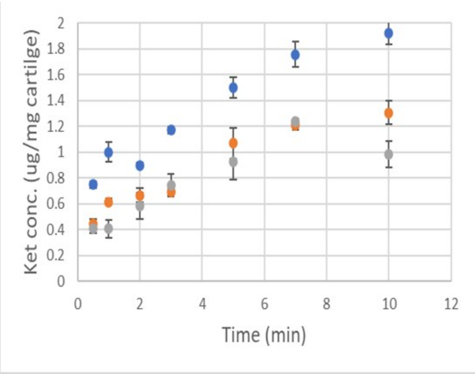
Ket conc. (ug/mg cartilge)
Time (min)

D5

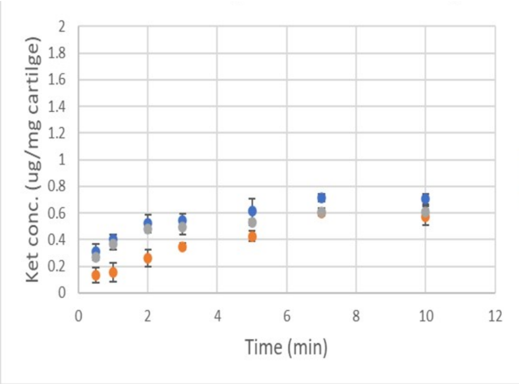
Ket conc. (ug/mg cartilge)
Time (min)

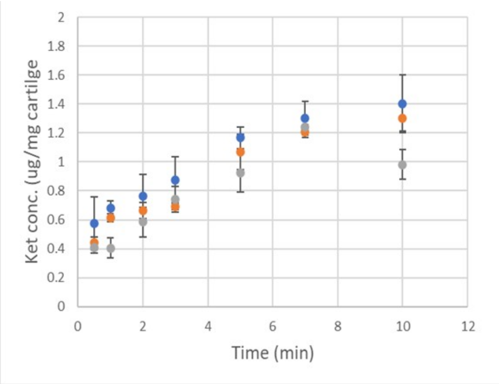
Ket conc. (ug/mg cartilge
Time (min)

E5

F5

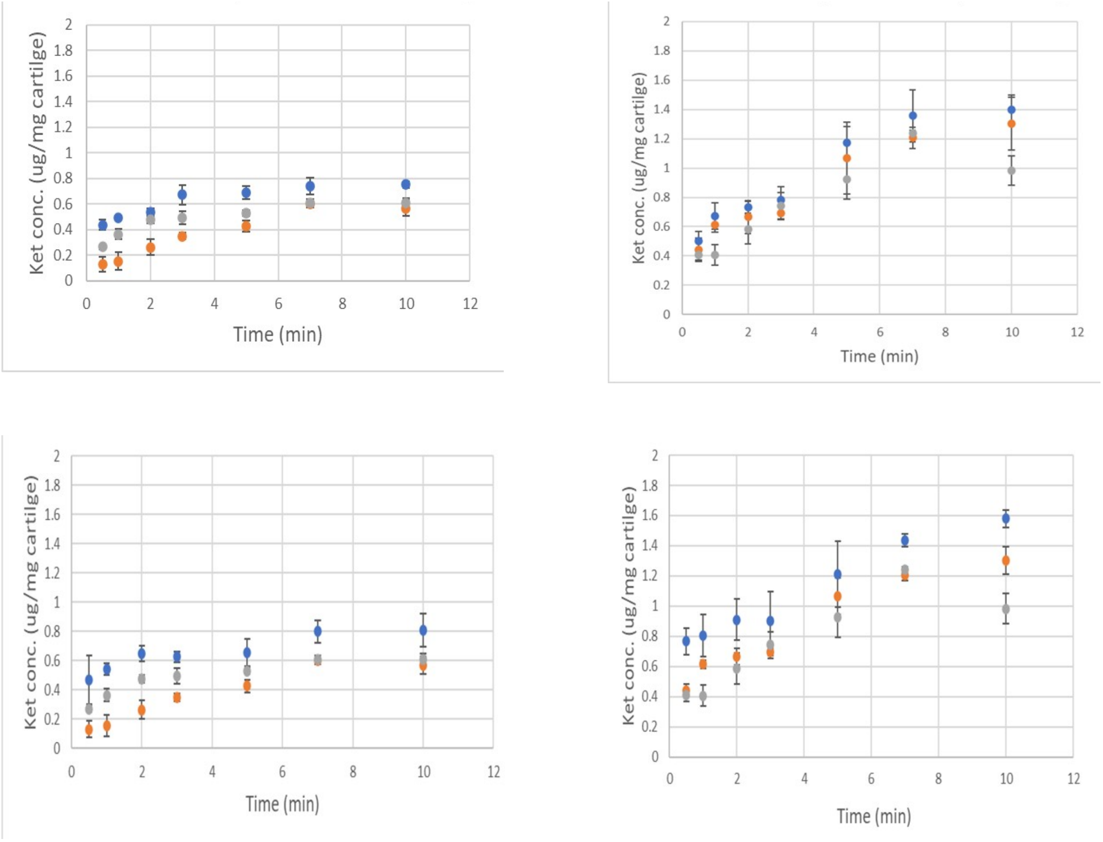

Figure S1: Comparison of ketorolac uptake into cartilage after 10 min exposure to (●) ketorolac o/w emulsion saturated with PBAE, (●) ketorolac in PBS, and (●) ketorolac o/w emulsion in both untreated and GAG depleted cartilage using different PBAEs.

Untreated cartilage tissue | **GAG depleted cartilage tissue**

A1

B1

D1

E1

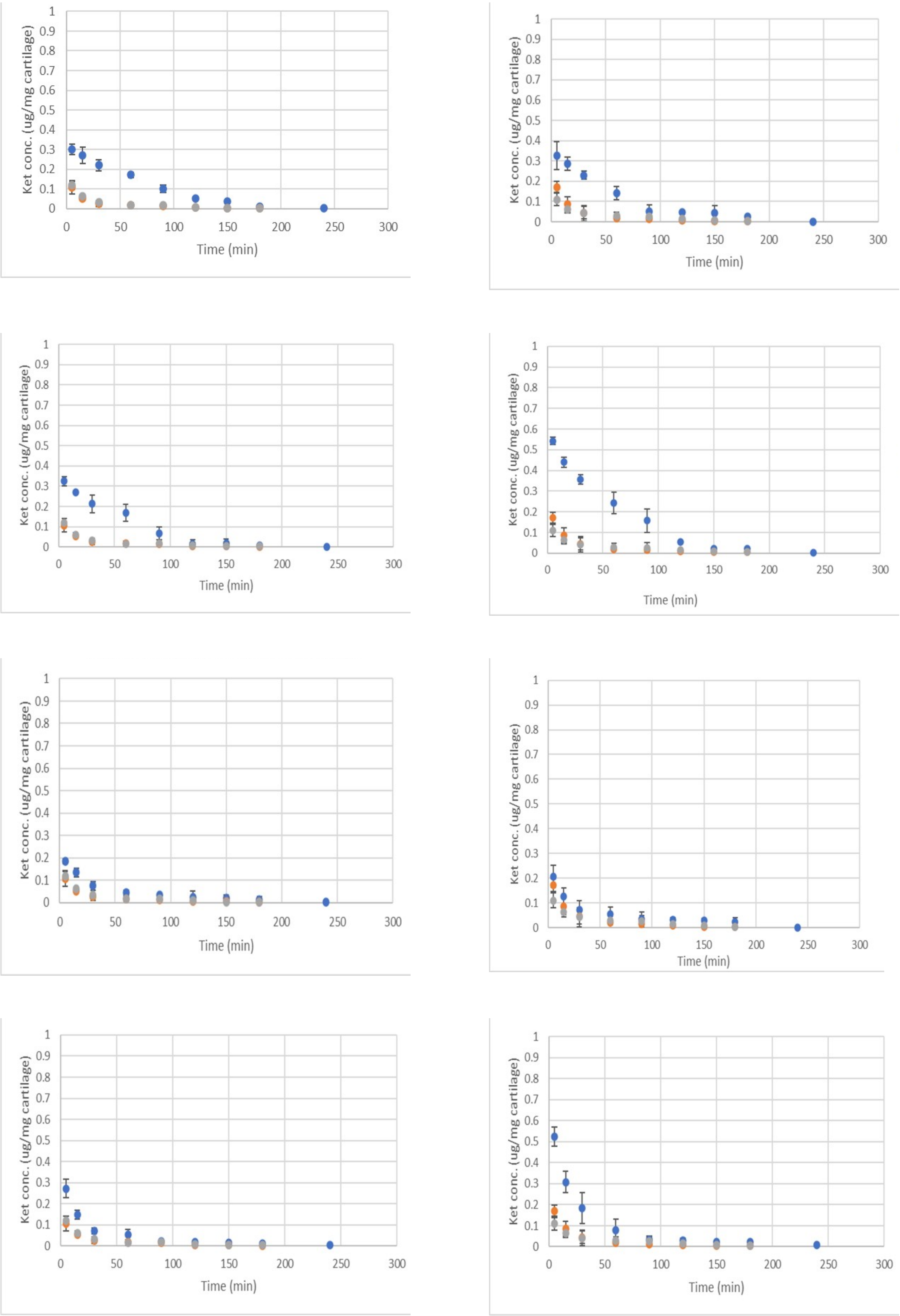

F1

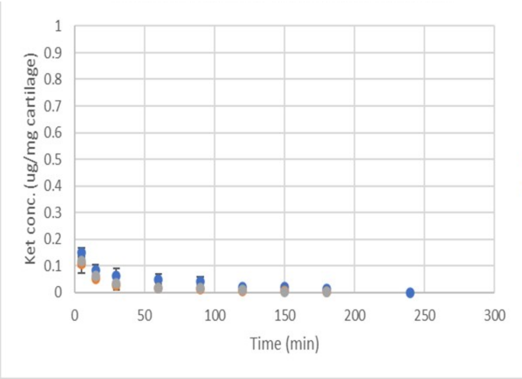
Ket conc. (ug/mg cartilage)
Time (min)

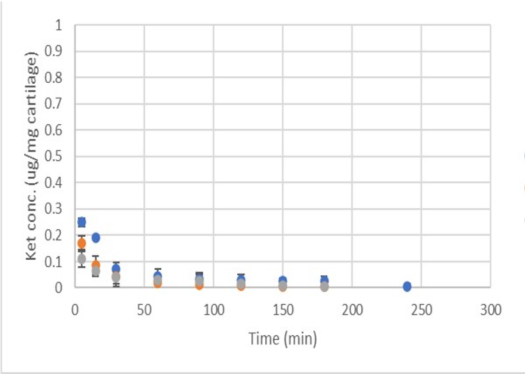
Ket conc. (ug/mg cartilage)
Time (min)

A3

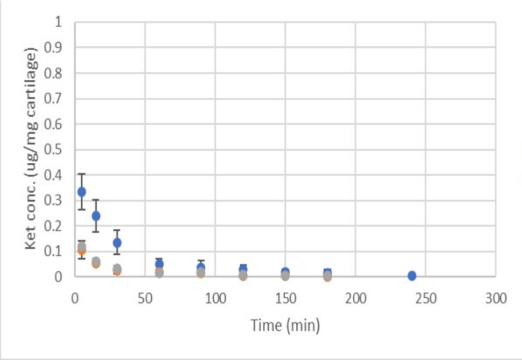
Ket conc. (ug/mg cartilage)
Time (min)

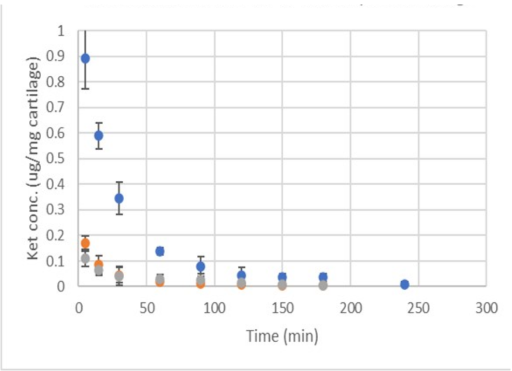
Ket conc. (ug/mg cartilage)
Time (min)

B3

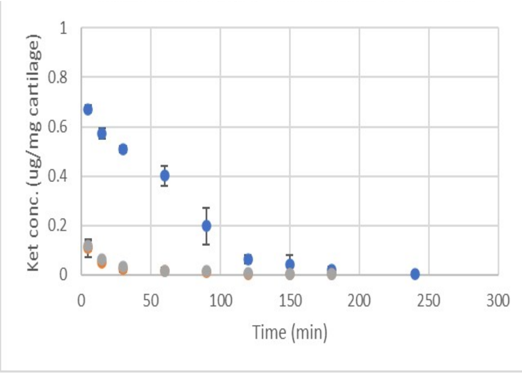
Ket conc. (ug/mg cartilage)
Time (min)

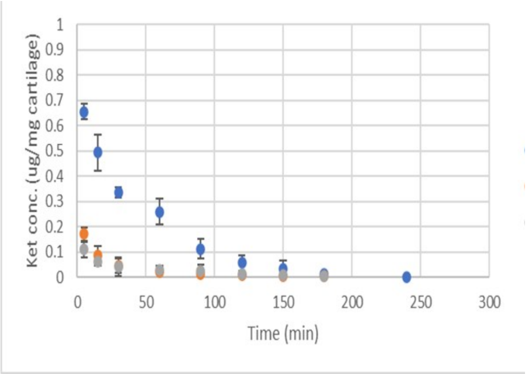
Ket conc. (ug/mg cartilage)
Time (min)

D3

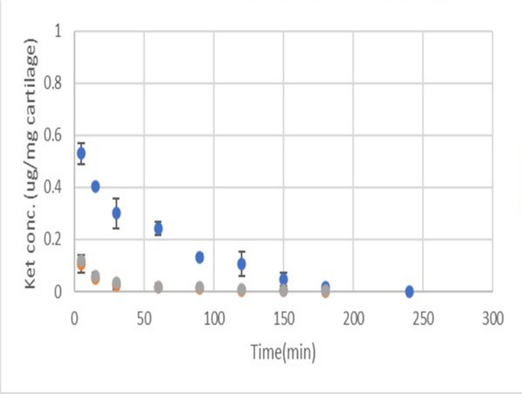
Ket conc. (ug/mg cartilage)
Time(min)

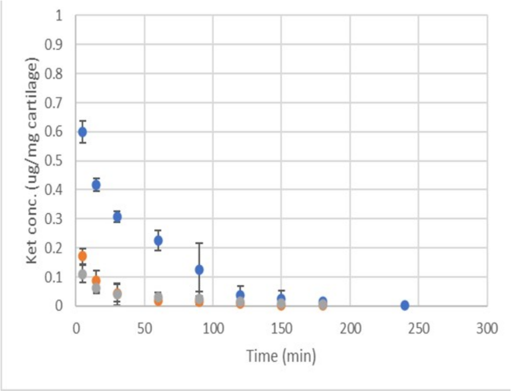
Ket conc. (ug/mg cartilage)
Time (min)

E3

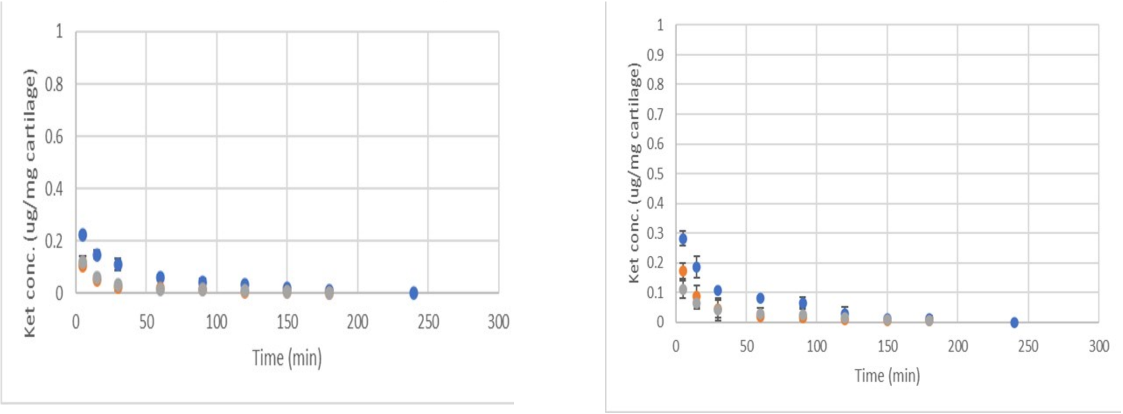
Ket conc. (ug/mg cartilage)
Time (min)
Ket conc. (ug/mg cartilage)
Time (min)

F3

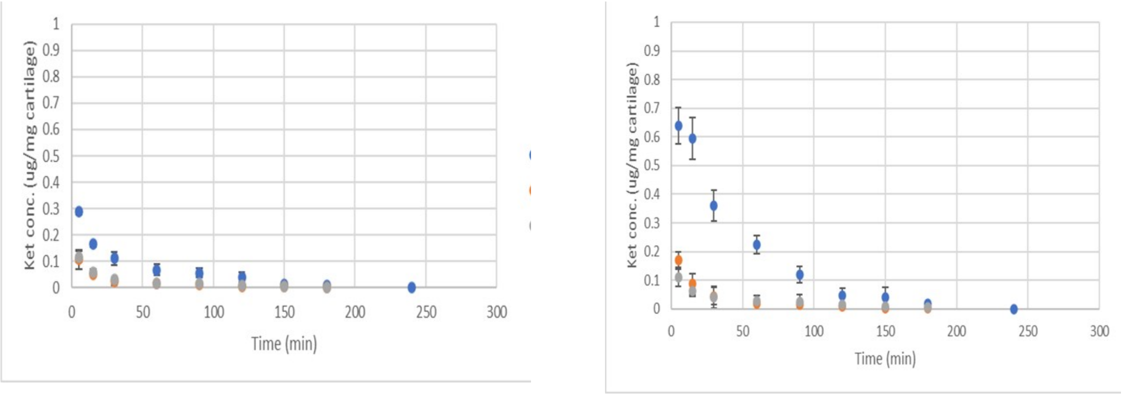
Ket conc. (ug/mg cartilage)
Time (min)
Ket conc. (ug/mg cartilage)
Time (min)

A5

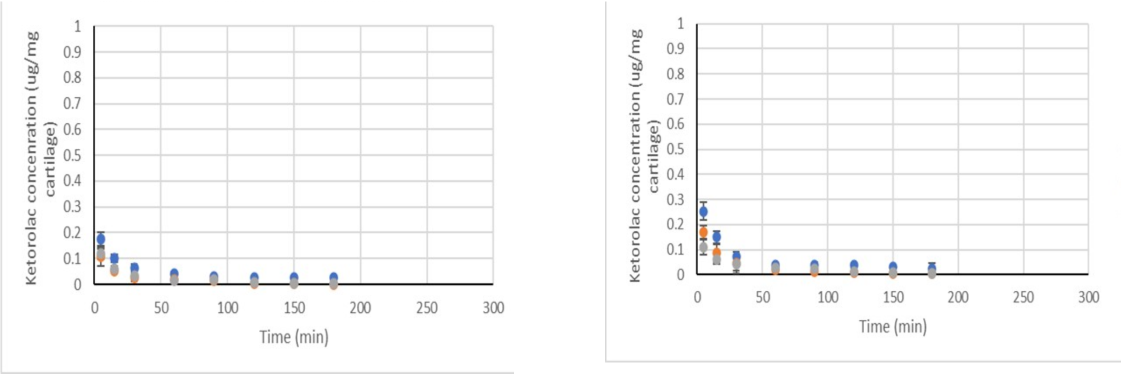
Ketorolac concenration (ug/mg cartilage)
Time (min)
Ketorolac concentration (ug/mg cartilage)
Time (min)

D5

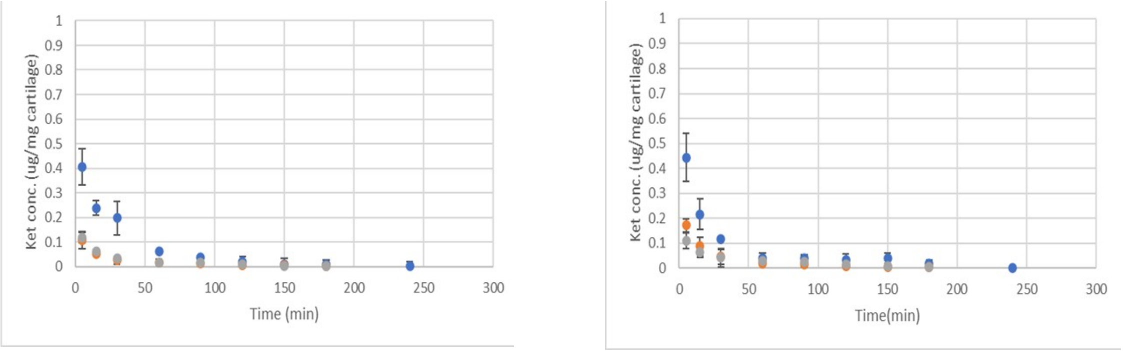
Ket conc. (ug/mg cartilage)
Time (min)
Ket conc. (ug/mg cartilage)
Time(min)

E5

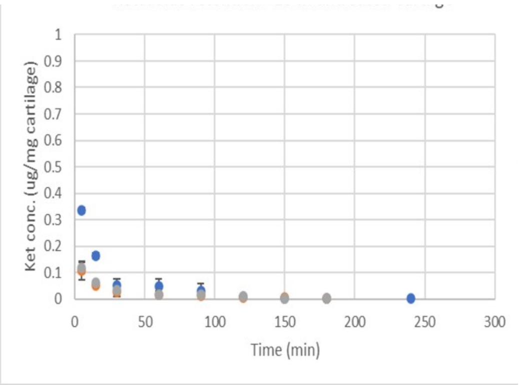

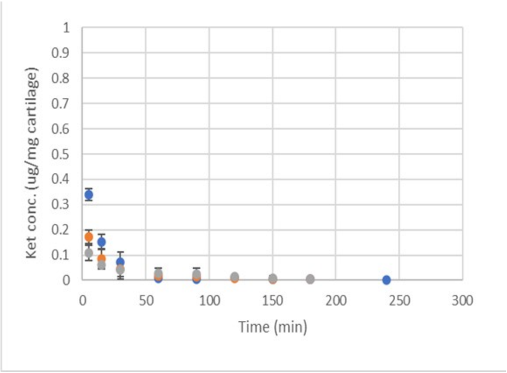

F5

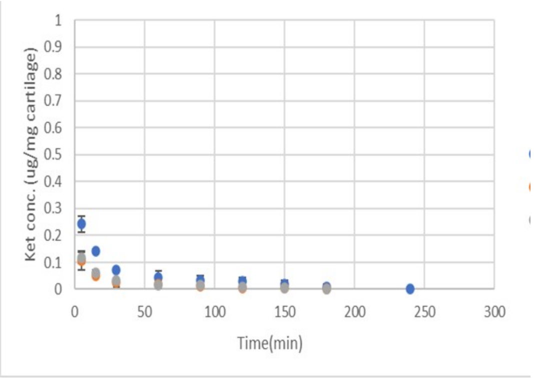

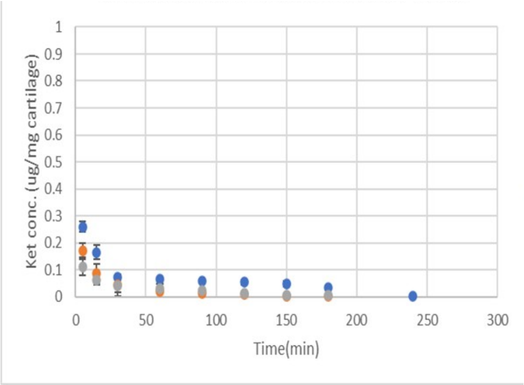

Figure S2: Comparison of ketorolac retention into cartilage for 300 min (5 hours) after 10 min exposure to (●) ketorolac o/w emulsion saturated with PBAE, (●) ketorolac in PBS, and (●) ketorolac o/w emulsion in both untreated and GAG depleted cartilage using different PBAEs.

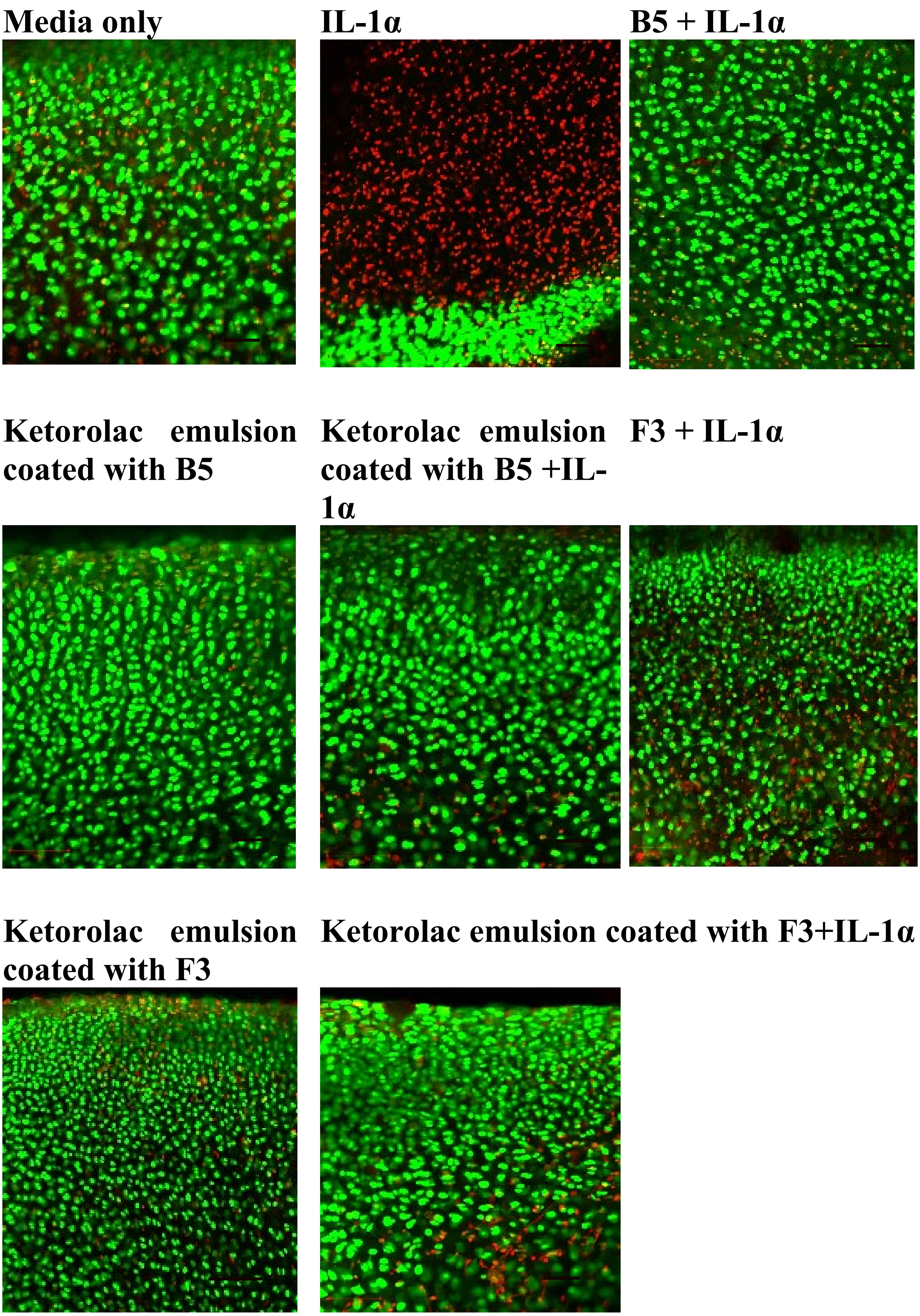

Figure S3 Examples of images of live/dead fluorescently stained sections of bovine cartilage explants exposed to IL-1α (1 ng/ml) and coated with F3 and B5 or uncoated ketorolac emulsions on Day 2. Live

cells were stained green by calcein, and dead cells were stained red by ethidium homodimer. Scale bar 100µm.
